# Potent anti-tumor activity of CD45RA-targeting Hm3A4-Ranpirnase against myeloid lineage leukemias

**DOI:** 10.1080/21655979.2022.2054159

**Published:** 2022-03-24

**Authors:** Sisi Li, Zhujun Wang, Xiaoping Guo, Ping Chen, Yongmin Tang

**Affiliations:** a School of Medicine, Zhejiang University City College, Hangzhou, Zhejiang, PR China; b Division/Center of Pediatric Hematology-Oncology at the Children’s Hospital of Zhejiang University School of Medicine, The Pediatric Leukemia Diagnostic and Therapeutic Technology Research Center of Zhejiang Province, National Clinical Medical Research Center for Child Health, Hangzhou, Zhejiang, PR China; c Department of Pediatrics, Union Hospital, Tongji Medical College, Huazhong University of Science and Technology, Wuhan, Hubei, PR, China

**Keywords:** Immunotoxin, ranpirnase, CD45RA, leukemic stem cells, Hm3A4, targeting therapy

## Abstract

CD45RA is a specific marker for leukemia stem cell (LSC) sub-populations in acute myeloid leukemia (AML). Ranpirnase (Rap), an amphibian RNase, has been extensively investigated in preclinical and clinical studies for its antitumor activity. Rap could be administered repeatedly to patients without inducing an immune response. Reversible renal toxicity has been reported to be dose-limiting. In this study, we generated a novel immunotoxin targeting LSCs: Hm3A4-Rap, which was composed of Rap and Hm3A4, a human-mouse chimeric antibody against CD45RA. This immunotoxin was generated recombinantly by fusing Rap to Hm3A4 at the Fc terminus and then produced by stably transfecting CHO cells. The immunotoxin was purified using Ni-NTA and then evaluated using RT-PCR, SDS-PAGE, antibody titer assays, competitive inhibition assays, and internalization assays. In addition, the purity, molecular integrity, and affinity to the CD45RA antigen were determined. *In vitro* studies demonstrated that Hm3A4-Rap could efficiently kill target cells. *In vivo* studies demonstrated that Hm3A4-Rap had potent anti-leukemia activity, with dosed mice showing a significant increase in survival time compared to control mice (P < 0.01). In summary, our immunotoxin had excellent biological activity suggesting its potential therapeutic value for treating AML patients. Additional preclinical and clinical studies are needed to develop this immunotoxin as a treatment option for patients with leukemia.

## Graphical Abstract

**Figure:**
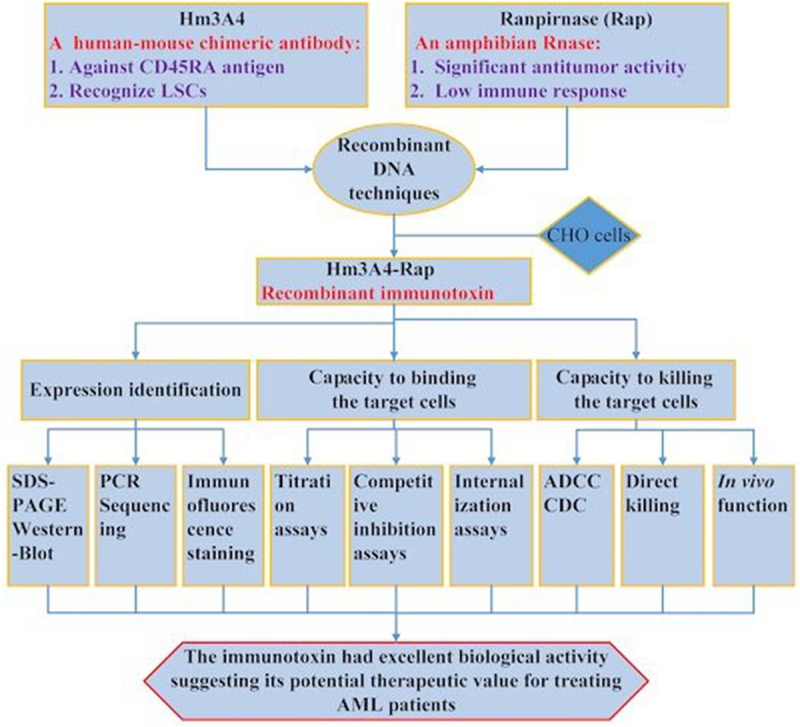

## 1. Introduction

Chemotherapy-resistant leukemic stem cells (LSCs) are thought to be responsible for relapses after therapy in acute myeloid leukemia (AML). CD45RA is expressed on leukemic cells in most AML patients. CD45RA has been used as a marker to specifically identify LSC and HSC and improve LSC quantification. Compared to other markers (CLL-1, also termed CLEC12A, CD33, and CD123), CD45RA is the most reliable marker [1–3]. In our previous study, we generated 3A4, a novel anti-CD45RA antibody where it could recognize CD45RA efficiently on target cells. Upon binding, 3A4 is rapidly internalized within 4 hours [4]. The norcantharidin (NCTD)-conjugated immunotoxin (NCTD-3A4) demonstrated potent antitumor activity in both *in vitro* and *in vivo* studies. In addition, we constructed a human-mouse chimeric antibody Hm3A4 by linking the murine ScFv3A4 to the human IgG1 Fc region. We found that the Hm3A4 antibody could eliminate leukemia cells including LSCs *in vitro* and *in vivo* [5]. Based on these results, we continued to perform additional development work on an immunotoxin based on Hm3A4.

Immunotoxins (ITs) are hybrid proteins comprising a targeting moiety of mostly single-chain Fv (ScFv) or Fab antibody fragments fused or chemically conjugated to a cytotoxic moiety [6,7]. The antibody moiety of the immunotoxin binds to tumor cells and then subsequently delivers the toxin into the cells to kill them [8,9]. ITs can be divided into three generation types: (i) first-generation ITs were produced by chemically conjugating native toxins to whole murine antibodies. These ITs had several drawbacks, i.e., they lacked specificity, had low stability, and a heterogeneous composition; (ii) second-generation ITs were fabricated by chemically conjugating a toxin fragment with no targeting activity following removal of the autonomic cell-binding domains to the murine antibody. This method had higher amounts of IT molecules that could be safely administered to animals and humans, but heterogeneity issues were still present; (iii) third generation ITs were produced using recombinant DNA techniques. These ITs, designated recombinant immunotoxins (RITs), mainly consisted of an ScFv fragment fused to a truncated toxin fragment [10–12].

Ranpirnase (Rap), a monomeric protein (*M*r, 11,817; 104 amino acids), is an amphibian ribonuclease (RNase) that belongs to the RNase A superfamily. Native Rap, isolated from *Rana pipiens* eggs [13], was demonstrated to have significant cytostatic and cytotoxic effects in a variety of tumor cell lines based on *in vitro* and *in vivo* studies [14,15]. They target precursor miRNAs to interfere with the assembly of tRNAs and down-regulate intracellular tumorigenic miRNA levels and have been demonstrated to kill tumor cells [16]. The molecular weight of Rap is approximately 22kDa. Unlike bacteria or phytotoxins, Rap has low immunogenicity and can be administered repeatedly to humans. Nonspecific toxic adverse reactions, such as vascular leakage syndrome, do not occur even at high doses due to their small molecular weight. The major side effects are nephrotoxicity, which most patients can tolerate, and can be reversed after drug cessation [17]. Rap has a low affinity (greater than 1 M) for RNase inhibitor (RI), which constitutes about 0.01% of the cytosolic protein in mammalian cells. Hence Rap can evade inactivation by RIs. Rap is highly stable and is resistant to both protease degradation and denaturation at elevated temperatures [18].

ImmunoRNAses are RNases that are linked to targeting antibodies or ligands. The immunoRNAse Rap (based on the frog RNase) tagged with a humanized, internalizing anti-Trop-2 antibody recognizes a cell surface glycoprotein that is overexpressed in a variety of epithelial cancers. It has been shown to induce potent cytotoxicity against several types of epithelial cancer cells [19]. Recently, an anti-human EGF receptor (-EGFR)-Rap fusion protein has shown specific anticancer activity for EGFR positive tumor cells [20]. An anti-CD22 conjugate between Rap and an IgG4-reformatted humanized version of the monoclonal antibody RFB4 showed significant *in vitro* cytotoxicity toward lymphoma and leukemia cell lines [21]. Rap has been investigated for the treatment of patients with unresectable malignant mesothelioma. Adverse renal dose-limiting toxicity was reversible with the absence of immunogenicity [22]. Although Rap is an amphibian protein, its small size, basicity, and homology to extracellular human pancreatic RNases result in very low immunogenicity in humans even after repeated administrations [23].

Based on the above findings, our hypothesis was that the recombinant Hm3A4-Rap immunotoxin can effectively kill the 3A4 positive AML cells, including leukemia stem cells (LSC). The aims of this study were to test the targeting activity of Hm3A4-Rap on AML cells and their LSCs both in vitro and in vivo. The goals of our work were to evaluate the biological activity of Hm3A4-Rap and provide a potential agent for AML therapy.

## 2. Materials and methods

### 2.1 Cell lines and cell culture

KG1a cells (human acute myelogenous leukemia cell line, purchased from ATCC, Manassas, VA) were cultured in IMDM medium with 20% (v/v) heat-inactivated fetal calf serum (FCS) [24]. Nalm-6 cells (human pre-B ALL cell line; kindly provided by

C Patrick Reynolds at the children’s hospital of Los Angeles) were cultured in PRMI-1640 medium with 10% (v/v) heat-inactivated fetal calf serum (FCS) [4]. CHO cells (purchased from ATCC, Manassas, VA) were cultured in RPMI 1640-Glutamax-I medium containing 10% (v/v) heat-inactivated FCS [5]. All cell lines were cultured in media supplemented with 100 units/mL of penicillin and 100 μg/mL of streptomycin (Sangon Bioengineering Co., Shanghai, China) at 37°C in a humidified 5% CO2 incubator.

### 2.2 Construction of the pHMCH3/Hm3A4-Rap expression vector

The pHMCH3/Hm3A4 plasmid was constructed in our laboratory as previously described [5]. The Rap gene was synthesized and inserted into the Xba1 and Apa1 restriction sites within the MCS of the plasmid. The Rap gene sequence was extracted from NCBI, optimized, and 3’ tagged with 6× His to enable subsequent purification [25]. Gene synthesis was performed by Sangon. cDNA for Rap was amplified using the following primers: forward:5’-GGCTCTAGACAGGACTGG-3’;reverse: 5’-GGCGGGCCCTTAGTGGTG-3’ and cloned into the pHMCH3/Hm3A4 vector to generate the pHMCH3/Hm3A4-Rap expression vector. The pHMCH3/Hm3A4 vector and amplified PCR product of the Rap gene were digested with Apa1 and Xba1 and then ligated. DNA sequences encoding each region including Fc, Fc-Rap, and ScFv3A4 were detected in transfected CHO cells using specific primers.

### 2.3 Transfection, expression, and purification of Hm3A4-Rap

The pHMCH3/Hm3A4-Rap plasmid was transfected into CHO cells using Lipofectamine™ 2000 (Invitrogen, Carlsbad, USA). Transfected cells were selected in serum-free medium (Invitrogen) containing 600 µg/ml of G418 (Sangon) to establish stable cell lines. After 2–3 weeks of selection, the supernatants of pHMCH3/Hm3A4-Rap plasmid transfected CHO cells were harvested and centrifuged at 6000 g for 10 min to remove cells and cell debris. The supernatants were then concentrated by ultrafiltration and purification using an immobilized nickel-iminodiacetic acid affinity chromatography column (Bio-Rad, California, USA) [26]. First, the pH of the solution was adjusted to 8.0 using 1 M KOH or 1 M H_3_PO4. The supernatants were then passed through the column that had been previously equilibrated with a wash buffer 1 (300 mM KCl, 50 mM KH_2_PO4, and 5 mM imidazole, pH 8.0). The column was washed with wash buffer 1 and then wash buffer 2 (300 mM KCl, 50 mM KH_2_PO4, and 10 mM imidazole, pH 8.0). Next, proteins on the column were eluted using elution buffer (300 mM KCl, 50 mM KH_2_PO4, and 250 mM imidazole, pH 8.0). The eluted fraction was collected and dialyzed against phosphate-buffered saline (PBS). Crude and purified culture supernatants were run on a 10% SDS–PAGE and analyzed by western blot using the horseradish peroxidase (HRP)-conjugated goat anti-human IgG Fc antibody (1:1000 dilution, Hua-an, Hangzhou, China). The concentration of Hm3A4-Rap protein was determined using the BCA kit (Beyotime, Haimen, China) [27].

### 2.4 Immunofluorescence staining

CHO cells expressing Hm3A4-Rap were grown on glass slides for approximately 24 h and then fixed in 4% paraformaldehyde, permeabilized with 0.2% Triton X-100, and blocked in PBS containing 10% calf serum. The cells were then stained with TRITC-conjugated anti-mouse Fab specific IgG antibody (T7782, diluted 1:200; Sigma) and FITC-conjugated goat anti-human IgG Fc antibody (GAHFc FITC) (Invitrogen) for 1 h in the dark. This was followed by incubation with DAPI (Invitrogen) nucleic acid (nuclear) staining for 1 min in the dark. The cells were visualized and imaged using confocal immunofluorescence microscopy (Carl Zeiss Scope.A1, Oberkochen, GER). Non-transfected CHO cells were used as the negative control. The expression of Hm3A4-Rap in CHO cells was detected by immunofluorescence microscopy. The ScFv3A4 fragment was detected using GAM-Fab-TRITC and the Fc fragment was detected using GAH-Fc-FITC [28].

### 2.5 Titration assays and competitive inhibition assays

To identify the binding capacity of Hm3A4-Rap, we performed titration assays. KG1a cells were harvested at their logarithmic growth phase and adjusted to a concentration of 10^7^/mL. 100 μL (10^6^ cells) of cell suspension was then incubated with various amounts (0.00125 µg, 0.0125 µg, 0.025 µg, 0.125 µg, 0.25 µg, 0.5 µg, 1.25 µg, 2.5 µg, and 5 µg) of purified Hm3A4-Rap for 30 min at 25°C in the dark. The cells were then incubated with fluorescein isothiocyanate (FITC)-labeled goat anti-human Fc (GAHFc-FITC, KPL, Gaithersburg, MD) for an additional 15 min at 25°C. Afterward, the cells were washed twice with PBS before flow cytometry analysis (FCM) (FACSCalibur™, Becton Dickson, San Jose, CA, USA). We also performed competitive inhibition assays to identify the relative binding affinity of Hm3A4-Rap in comparison with the parental antibody, 3A4 mAb. Briefly, KG1a cells were incubated with 3A4-FITC at a fixed sub-saturated concentration of 0.5 µg, either alone or in the presence of varying amounts of Hm3A4-Rap (0.00125 µg, 0.0125 µg, 0.025 µg, 0.125 µg, 0.25 µg, 0.5 µg, 1.25 µg, 2.5 µg, and 5 µg) for 30 min at 25°C. The same amounts of mouse IgG1 isotype antibody (Hua-an, Hangzhou, China) was used as the control. After three washes with PBS, stained cells were analyzed by FCM. Experiments were performed in triplicates.

### 2.6 Internalization assay

Internalization of the Hm3A4-Rap antibody in KG1a cells was determined and compared to that of the Hm3A4 antibody. 0.1 mol/L glycine (pH = 2.5) was used to remove unbound antibodies on the surface of KG1a cells [29]. Cells were divided into the experimental and control group. 5 × 10^5^ of KG1a cells were incubated with 1 μg of Hm3A4-Rap or Hm3A4 at 37° for 0.5 h, 1 h, 2 h, or 4 h. Then, 0.1 mol/L glycine (pH = 2.5) was added to the experiment tube to remove any unbound antibody on the surface of KG1a cells and then washed twice with PBS containing 1% BSA. Cells in the control group were washed with PBS containing 1% BSA to remove unbound antibodies. Cells were then resuspended and fixed with 4% paraformaldehyde for 10 min, then permeabilized with 0.2% Triton X-100 for 5 min and incubated with 2 μL of GAH-Fc-FITC antibody for 30 min. Cells were then washed twice with PBS, resuspended in PBS, and analyzed by FCM. Experiments were performed in triplicates.

$$\%\;Degree\;of\;internalization=\frac{Positive\;rate\;of\;Control-Positive\;rate\;of\;Experiment}{PositiverateofControl}\times 100$$

### 2.7 Targeting of Hm3A4-Rap to KG1a cells

ADCC function was evaluated using the CytoTox 96 ® Non-Isotopic Cell Killing Test Kit (Promega, Madison, Wisconsin, USA). Briefly, peripheral blood mononuclear cells (PBMC) were isolated from the blood of healthy volunteer by Ficoll-Hypaque separation (GE Healthcare/Amersham Biosciences) to be used as effector cells. Cells were washed twice with Hanks Balanced Salt Solution (Invitrogen) to remove platelets and then resuspended in RPMI-1640 media supplemented with 10% heat-inactivated fetal bovine serum (HIBS) (5 × 10^6^ cells/mL).

The following assay conditions were set up: 1) Effector spontaneous wells; 2) Target spontaneous wells; 3) Target Maximum wells (Target cells with 0.8% Triton X-100; and 4) Experimental wells (effector cells with target cells and Hm3A4-Rap or Hm3A4). Three replicate wells were set for each condition. Step1: Hm3A4-Rap and Hm3A4 antibodies were diluted to a concentration of 0.1 mg/mL using phenol red-free RPMI-1640 media. Step2: Target cells were added and incubated at 4°C for 30 min. Cells were then washed twice with PBS and resuspended in 300 μL RPMI-l640 media. Step3: Cell suspensions with antibodies were added to a 96-well plate at 100 μL/well. Step4: 100 μL/well of effector cells were added at a ratio of 25:1 or 50:1 KG1a cells. Cells were then placed in a 5% CO2 incubator for 12 h to 24 h. Step5: Cells were centrifuged and then 50 μL of the supernatant was transferred from each well to a new 96-well plate. The absorbance at 490 nm of each well was measured using the Epoch 2 Microplate Spectrophotometer (BioTek, Winooski, VT, USA). Cytotoxicity was calculated based on the following formula:

$$\%Cytotoxicity=\frac{Experimental-Effector\;Spontaneous-Target\;Spontaneous}{Target\;Maximum-Target\;Spontaneous}\times 100$$

Complement-dependent cytotoxicity (CDC) assay was performed using the Cell Counting Kit-8 (CCK-8) method, which is a rapid, reliable, and sensitive measurement for cell viability, details of which have been described previously [5]. Briefly, KG1a cells were washed and resuspended in serum-free RPMI-1640 at a concentration of 1 × 10^6^ cells/mL. 50 μL of the cell suspension was added to each well of a 96-well plate, and then 25 μL of different concentrations of Hm3A4-Rap and Hm3A4 were added to each well and incubated for 1 h. 25 μL of human serum as a complement source from healthy volunteers was added per well and incubated at 37°C for 2 h. Wells with no antibodies containing equal volumes of culture medium and complement was used as the negative controls. Three replicate wells were performed for each condition. 10 μL of CCK-8 (Beyotime Institute of Biotechnology) was added to each well and incubated for an additional 2 h at 37°C. The absorbance at 450 nm of the formazan dye produced by metabolically active cells from each well was measured using the Epoch 2 Microplate Spectrophotometer. Cytotoxicity was calculated based on the following formula..

$$\%\;cytotoxicity=\left(1-\frac{T-C}{E-C}\right)\times 100$$

Where E was the absorbance of the medium group, C was the absorbance of the complete lysis group, and T was the absorbance of the experimental group.

Direct killing effect of Hm3A4-Rap on KG1a: 1) We compared the cytotoxic effects of Hm3A4-Rap with Hm3A4 using Annexin-V/PI Kit(Beyotime Institute of Biotechnology). The concentration of Hm3A4 and Hm3A4 were both set at 2.5 μg/mL, 5 μg/mL and 10 μg/mL in 4 mL volume/well of a 6-well plate. Three replicate wells were performed for each condition. Cells were incubated for 48 h at 37°C, 5% CO2. Then, cells were washed twice with ice-cold PBS and centrifuge for 5 minutes at 300 g at 4°C. Discard supernatant, and resuspend the cells in ice-cold binding buffer to 1 × 10^6^ cells/mL. Add 5 μL of Annexin V-FITC and 5 μL of PI to 100 μL of the cell suspensions. Mix gently and keep tubes on ice and incubate for 15 minutes in the dark. Then, add 400 μL of ice-cold binding buffer and mix gently. Analyze cell preparations within 30 minutes by FCM. The Annexin+/PI- and Annexin+/PI+ cells were identified as apoptotic and necrotic cells. 2) The cytotoxic effect of Hm3A4-Rap or 3A4 on KG1a or Nalm-6 cells was measured using the traditional cell counting method. The concentration of Hm3A4-Rap and 3A4 were both set at 10 μg/mL and 20 μg/mL in 2 mL volume/well of a 24-well plate. Control wells were treated with equal volumes of PBS. Three replicate wells were performed for each condition. Cells were incubated for 24, 48, 72, and 96 h at 37°C, 5% CO2. Cell numbers were measured using Countstar (Shanghai Zeyu Experimental Equipment Co. Ltd, Shanghai, China). Cytotoxicity was calculated based on the following formula:

$$\%cytotoxicity=\frac{Cell\;number\;of\;PBS-Cell\;number\;of\;Experiment}{Cell\;number\;of\;PBS}\times 100$$

### 2.8 In vivo antitumor activity in NOD/SCID mice

To determine the *in vivo* antitumor activity of Hm3A4-Rap, we established a xenograft model by inoculating KG1a cells into non-obese diabetic (NOD)/severe combined immunodeficiency (SCID) mice (animal study protocol number 2013-GJ-010). The model was established as follows: Twenty specific pathogen-free 4-week-old female NOD/SCID mice (Zhejiang University, Hangzhou, China) were used for model generation and were divided into four groups with 5 mice for each group (n = 5): (1) the control group, (2) PBS treatment, (3) Hm3A4-Rap treatment, and (4) Hm3A4 treatment group. Mice were administered cyclophosphamide (CTX) by intraperitoneal injection 1 day before xenografting and were randomly assigned to the control or the xenograft group. The xenograft group was further subdivided into the PBS, Hm3A4-Rap, and Hm3A4 treatment groups.

On day 0 of the study, 1 × 10^6^ exponentially growing KG1a cells in 100 μL or an equal volume of PBS were tail vein injected into the xenograft or the control group, respectively. Mice in the xenograft group were treated intravenously with PBS, Hm3A4-Rap, or Hm3A4 on d1, d4, and D7, respectively. Disease symptoms (ruffled coat, hunched back, weakness, and reduced motility) were monitored daily. Mice with disease symptoms were euthanized by cervical dislocation.

### 2.9. Statistics

*In vitro* data were analyzed using a t-test. *In vivo* data between the groups was analyzed using the Kaplan–Meier method. P < 0.05 was considered statistically significant.

## 3. Results

We generated the Hm3A4-Rap immunotoxin using recombinant DNA techniques. The potential therapeutic efficacy of the immunotoxin Hm3A4-Rap on target cells was evaluated for its binding capacity, internalization function, and most importantly the ability to kill target cells.

### 3.1 Construction and transfection of pHMCH3/Hm3A4-Rap

All PCR products were verified to be correct in size and sequence (Supplementary file). Sangon sequencing demonstrated no mutations in the Hm3A4-Rap gene and was of the correct size, i.e., 7181bp. We are still in the patent application stage, so we cannot publish all sequences, please understand.

### 3.2 Expression of pHMCH3/Hm3A4-Rap in CHO cells

**Figure 1.**
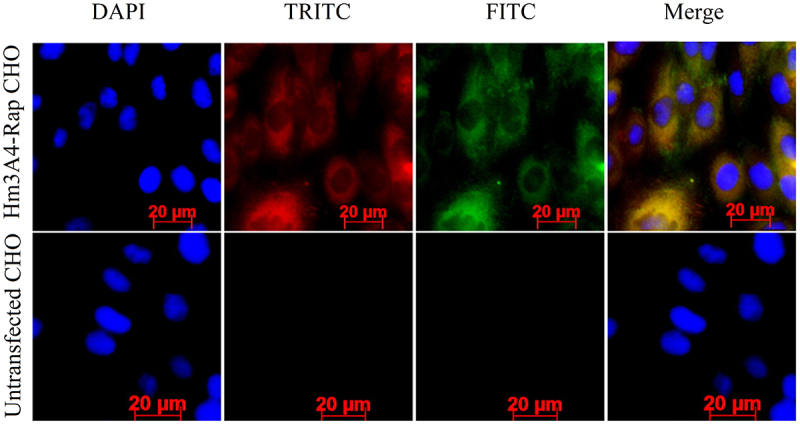
Expression of recombinant proteins in the cytoplasm of Hm3A4-Rap CHO cells was measured using immunofluorescence microscopy. DAPI: labeled nuclei; TRITC: labeled murine Fab fragments; FITC: labeled human Fc fragments; Merge: superimposed images. In CHO cells transfected with the target plasmid, we can see that both TRITC and FITC fluorescence appear in the cytoplasm, which are shown as yellow fluorescence in the merge map, with a positive rate of 18/19. No TRITC or FITC fluorescence was observed in Non-transfected CHO cells.

Immunofluorescence microscopy for red and green fluorescence was detected in the cytoplasm of CHO/Hm3A4-Rap cells. Images were superimposed, in which, yellow images indicated colocalization indicating that CHO/Hm3A4-Rap cells successfully expressed the recombinant protein. No FITC and TRITC fluorescence was observed in transfected CHO cells (Figure 1).

### 3.3 Binding capacity and internalization of the fusion antibody

**Figure 2.**
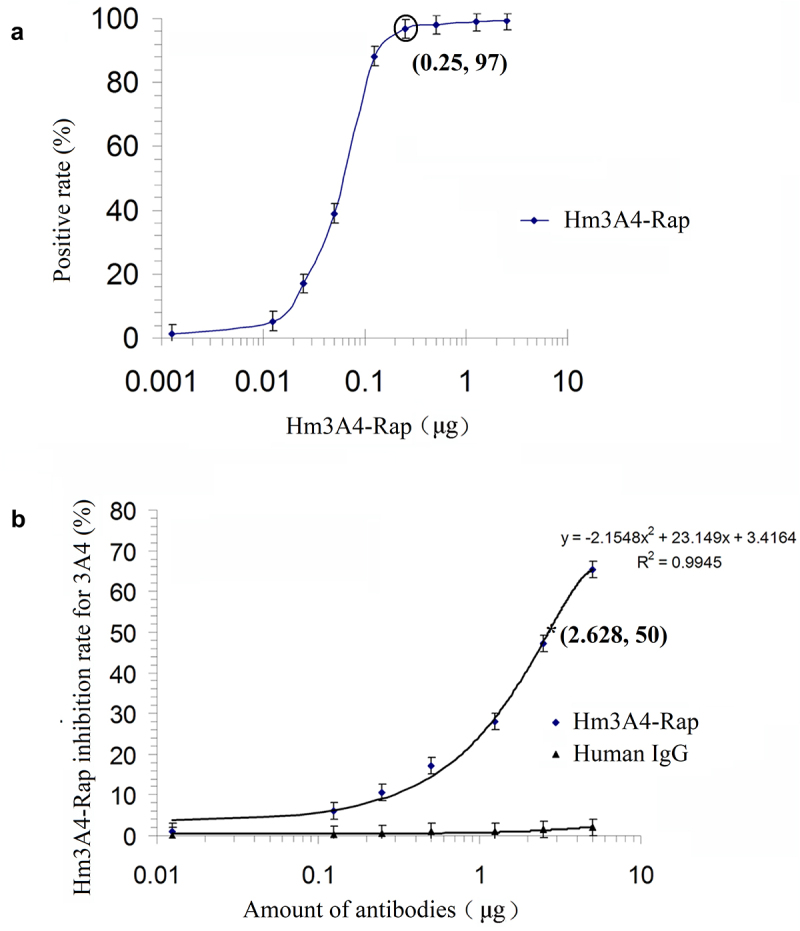
Antibody titration of Hm3A4-Rap (a) competitive binding assay results for 3A4-FITC (n = 3) (b). As the concentration of scFv3A4-FcRap antibody increased, its binding to antigen gradually reached saturation (n = 3). When 0.25 µg of scFv3A4-FcRap antibody reacts with 1 × 10^6^KG1a cells, the positive cell rate can reach more than 97% (a). Competitive binding experiments showed that when the amount of antigen (number of cells) was fixed, as the amount of Hm3A4-Rap increased, the binding of 3A4 to the antigen gradually decreased (b).

300 mL of CHO/Hm3A4-Rap cell culture supernatant was harvested and then concentrated by ultrafiltration to obtain 3 mL (100:1) of concentrated supernatant. 15 mL of the purified antibody were collected and concentrated to 5:1 using ultrafiltration to a final concentration of 1 mg/mL. The amount of Hm3A4-Rap secreted in the CHO/Hm3A4-Rap cell culture supernatant was estimated to be 10 μg/mL. Titration results indicated that 0.25 μg of Hm3A4-Rap resulted in 97% positivity in 10^6^ KG1a cells (Figure 2). Competitive binding studies demonstrated that when the antigen concentration was fixed, and the amount of Hm3A4-Rap was gradually increased, a gradual decrease in 3A4 antibody binding to the antigen in KG1a cells was observed. The percentage decrease in the 3A4 antigen-binding rate induced by 0.0125 μg, 0.125 μg, 0.25 μg, 0.5 μg, 1.25 μg, 2.5 μg and 5 μg of Hm3A4-Rap was 1.25%, 6.3%, 10.65%, 17.19%, 28.04%, 47.87%, and 65.85%, respectively. The regression curve was, y = −2.1548x^2^ + 23.149x + 3.4164 (where, x was the amount of antibody and y was the antibody antigen-binding inhibition rate, R^2^ = 0.9945). The antigen-antibody binding decreased by 50% when Hm3A4-Rap was 2.682 μg (Figure 2). The protein molecular weight was approximately 135kDa (dimer) based on the amino acid sequence of the Hm3A4-Rap antibody. 2.682 μg was calculated to be approximately 1.98 × 10^−11^M, and the relative affinity constant (reciprocal of the mole number) was Kd = 5.0 × 10^10^M^−1^.

**Figure 3.**
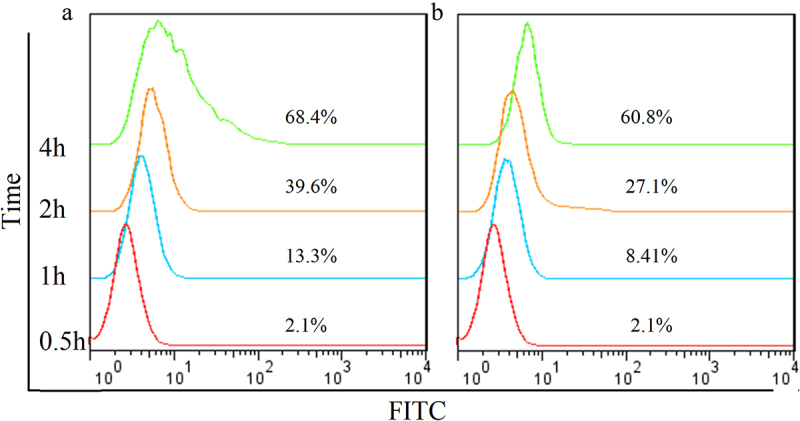
Internalization rate analysis of Hm3A4-Rap (a) and Hm3A4 (b) determined by FCM. Gradual internalization into cells was observed with prolonged incubation times, there was no significant difference in the internalization rate between the two (P > 0.05).

Internalization studies demonstrated that the degree of internalization of Hm3A4-Rap at different time points were 2.1% (0.5 h), 13.3% (1 h), 39.6% (2 h), and 68.4% (4 h), while they were 2.1% (0.5 h), 8.41% (1 h), 27.1% (2 h), and 60.8% (4 h) for Hm3A4. These results indicated that Hm3A4-Rap could be efficiently internalized into cells with prolonged incubation times. The internalization degree was similar to Hm3A4 (Figure 3).

### 3.4 In vitro *antitumor activity of Hm3A4-Rap*

**Figure 4.**
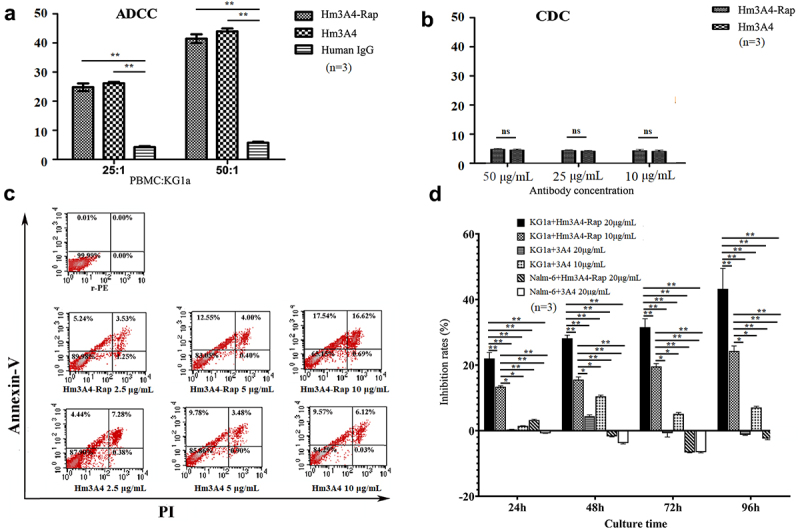
Antitumor activity of Hm3A4-Rap. ADCC function (a): When the ratio of PBMCs to KG1a was 25:1, the mean lysis rate of KG1a in the Hm3A4-Rap group was 24.8% vs. 4.23% for the IgG group (p < 0.05); when the ratio was increased to 50:1, the mean lysis rate was 41.47% vs. 5.73% for the IgG group (p < 0.05). When the ratio of PBMCs to KG1a was 25:1, the mean lysis rate of KG1a in the Hm3A4 group was 26.16% vs. 4.23% for the IgG group (p < 0.05); when the ratio was increased to 50:1, the mean lysis rate was 43.98% vs. 5.73% (p < 0.05). CDC function (b): Different concentrations of Hm3A4-Rap and Hm3A4 had no significant effect on the CDC in KG1a cells. (c) The cytotoxicity of Hm3A4-Rap and Hm3A4 on KG1a cells was detected by flow cytometry Annexin-V/PI method. (d) Effects of Hm3A4-Rap or 3A4 on the proliferation of KG1a cells or Nalm-6 cells. Hm3A4-Rap significantly inhibited the proliferation of KG1a cells.

Both Hm3A4-Rap and Hm3A4 were able to effectively kill CD45RA-positive KG1a cells as determined by ADCC and were enhanced with increased ratios of PBMC: KG1a. Hm3A4-Rap (compared with human IgG, the mean cell lysis rate was 24.8% vs. 4.23% when PBMC:KG1a = 25:1, p < 0.05; when PBMC:KG1a = 50:1, the mean cell lysis rate was 41.47% vs. 5.73%, p < 0.05) and Hm3A4 (compared with human IgG, when PBMC:KG1a = 25:1, the mean cell lysis rate was 26.16% vs. 4.23%, p < 0.05; PBMC:KG1a = 50:1, the average cell lysis rate was 43.98% vs. 5.73%, p < 0.05). The ADCC of Hm3A4-Rap was comparable to that of the Hm3A4 antibody (p > 0.05) (Figure 4). The CDC activities of Hm3A4-Rap and Hm3A4 were not significant (Figure 4). The results of direct killing demonstrated the killing rate of 10 μg/mL Hm3A4-Rap for 48 h was 34.16%, and the corresponding Hm3A4 was 15.69% (Figure 4). With increasing Hm3A4-Rap incubation times ranging from 24 h to 96 h, the percent inhibition of KG1a cells by 10 μg/mL and 20 μg/mL of Hm3A4-Rap increased from 13.72% to 24.53% and 22.02% to 43.24%, respectively and the cytocidal activity was time and dose-dependent (P < 0.01) (Figure 4).

### 3.5 In vivo *antitumor activity of Hm3A4-Rap*

**Figure 5.**
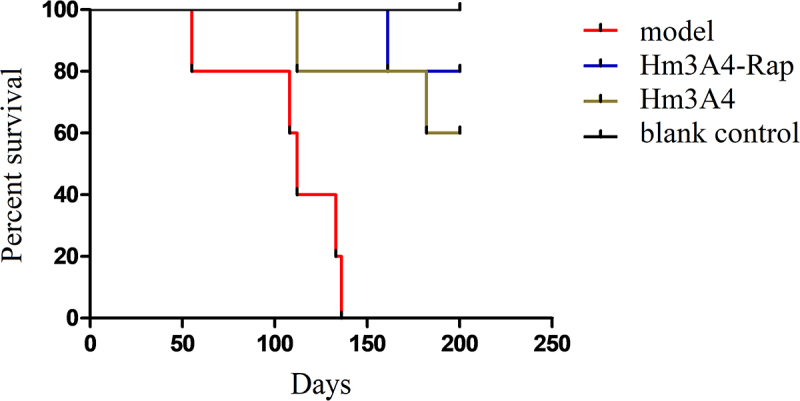
Survival analysis of the treated and control xenograft mouse model groups. After the 136th day, all NOD/SCID mice in the modeling group died, while one of the Hm3A4-Rap-treated mice died at 165 days, and the remaining 4 survived well (p < 0.05 compared with the modeling group); Hm3A4-treated mice had one death at 112 days, another at 182 days, and the remaining three survived well, and the difference was statistically significant compared with the model group (p < 0.05).

We observed a significant reduction in overall survival in the model (PBS treatment) group compared to the Hm3A4-Rap treatment group. Mice in the model group died on the following days: day55, day108, day112, day134, and day136. For mice in the Hm3A4-Rap treated group, one mouse died at day165, and the remaining four survived with no disease symptoms. For mice in the Hm3A4 treated group, one mouse died at day112, and another died at day182, while the remaining three mice survived with no disease symptoms. Both groups were statistically significant compared to the control group (p < 0.05). We extended the observation time to 200 days, during which, all remaining mice survived (Figure 5).

## 4. Discussion

LSCs are the root cause of leukemia relapse and drug resistance. The development of antibodies that can recognize LSCs is of great significance for AML treatment. At present, studies performed on LSCs are limited. KG1a cells are myeloid leukemia cell lines, a subset of cells with tumor stem cell-like biological characteristics. They express CD34 (98%) and CD123 (90%), and some do not express CD38, which is consistent with the currently recognized immunophenotypic characteristics of LSCs. Consistent with this, KG1a cells could be used as LSCs for research studies. In our study, we use KG1a cells for *in vitro* and *in vivo* studies.

Titration and competitive binding studies demonstrated that Hm3A4-Rap could effectively bind to the target antigen and inhibit the binding of the 3A4 antibody to antigen. Ka was found to be 5.0 × 10^10^M^−1^, indicating that it had good binding capacity and affinity. ADCC of Hm3A4-Rap was comparable to that of the Hm3A4 antibody, and significantly higher compared to IgG. This indicated that the fusion of Rap with the Fc fragment did not affect the binding of the Fc fragment to effector cells, which is an important mechanism for antibody-based targeting therapy [30]. CDC studies demonstrated both Hm3A4-Rap and Hm3A4 could not kill target cells via CDC function. This was consistent with our previous results that the antibody could not activate complement to initiate CDC function. It is worth noting that in CDC experiment, the incubation time was 2 hours. Although Hm3A4-Rap could be partially (39.6%) internalized into the cytoplasm, it was not enough for Hm3A4-Rap to play a role in killing cells. However, in direct killing experiment, when we prolonged the incubation time, it showed a clear killing effect. *In vivo* studies showed that the survival time in mice treated by Hm3A4-Rap was significantly longer than those treated by either Hm3A4 and PBS treated mice. The 150-day and 200-day survival rates of the Hm3A4-Rap group were 100% and 80%, respectively, and those of the Hm3A4 group were 80% and 60%, respectively. The death time of the first dead mouse in the Hm3A4-Rap group was 53 days later than that in the Hm3A4 group. Although there is no statistical difference between Hm3A4-Rap group and Hm3A4 group due to the small number of mice, a clear trend can be found. And finally, the immunotoxin generated by the fusion of Rap and the Hm3A4 antibody increased the molecular weight of Hm3A4 and prolonged its *in vivo* half-life [31]. Therefore, we believe that Hm3A4-Rap has a stronger antitumor activity compared to Hm3A4 and 3A4. We did not compare the killing effect of Hm3A4-RAP and RAP for the following reasons: we could not purchase the purified Rap, and the preparation of purified RAP requires very large engineering; moreover, our main purpose is to compare the killing effect of the immunotoxins and the plain antibody.

Regarding MAb-targeting drugs, intracellular transport, or internalization of the conjugated MAb is believed to be one of the major mechanisms responsible for tumor cell killing. Our results demonstrated that Hm3A4-Rap could be internalized into cells rapidly after binding to the target antigen in a time-dependent manner. The result was based on a single cell line KG1a and but if we can find a chance to test more cell lines in the future, the results will be more meaningful.

The direct killing effect of Hm3A4-Rap was not as high as expected. In our previous study, we generated Norcantharidin-conjugated 3A4 (3A4-NCTD) using the active ester method. The inhibition percentage of KG1a cells by 3A4-NCTD was 61.10% after 96 h of co-culture [4], while it was only 43.24% in Hm3A4-Rap. Dr. Nooshin Taghizadegan engineered the Rana pipiens RNase to bind to the ScFv of human anti-epidermal growth factor receptor (EGFR) antibody. The immunotoxin functions assessed in A431 cancer cells and EGFR‐negative HEK293 cells were found to have IC50 values of 22.4 ± 3 and >620.4 ± 5 nM, respectively. To enhance the killing efficacy of the Hm3A4-Rap fusion protein, improved methods should be considered based on the publication by Dr. Yu et al. [32] They developed an immunotoxin that targeted HER2. Their immunotoxin consisted of a partial translocation domain of Pseudomonas exotoxin, which was found to promote the release of toxins into the cytosol of gastric cancer cells and induce over 80% cell death. It could also effectively inhibit the growth of tumors in nude mice and significantly prolong their survival.

Regarding the structure of the immunotoxin, we believe that the 6× His-Tag epitope may interfere with the immune response. For future studies, we may consider making changes to the immunotoxin to that of N`-Rap-ScFv-Fc-C` [33]. Purification could be performed using Protein A columns as it binds to the Fc segment [34].

## 5. Conclusion

We have successfully generated the fusion protein Hm3A4-Rap using recombinant DNA techniques to target leukemia cells. The fusion protein retains the affinity and the antigen recognition specificity as its parental antibody and has the toxicity of Rap. Furthermore, the immunotoxin can kill target cells through ADCC. *In vitro* and *in vivo* studies have showed that Hm3A4-Rap has a good antitumor effect. We believe that the immunotoxin developed by us is an effective therapeutic candidate for clinical development.
